# Reproduction of sediment deposition and prediction of ^137^Cs concentration in the major urban rivers of Tokyo

**DOI:** 10.1038/s41598-020-65700-y

**Published:** 2020-06-12

**Authors:** Goro Mouri

**Affiliations:** Earth Observation Data Integration & Fusion Research Initiative (EDITORIA), The University of Tokyo, Be505, 4-6-1 Komaba, Meguro-ku, Tokyo 153-8505 Japan

**Keywords:** Environmental sciences, Environmental social sciences, Hydrology, Natural hazards

## Abstract

Radioactive caesium- 137 (^137^Cs) can be used as a tracer to infer sediment dynamics due not only to its long radioactive half-life but also its affinity for fine sediment. A novel advanced interpolation assessment was conducted to examine radionuclide activity in terraced land covered with volcanic ash soil in Tokyo, Japan, which had a time-dependent input function and incorporated the effects of mixed-sediment particle dynamic behaviour on radioactive decay. In addition, transport parameters derived from Chernobyl measurements were applied as predictors of the long-term contamination of the cardinal urban rivers by the fallout from the Tokyo Electric Power Fukushima Daiichi Nuclear Power Plant (FDNPP) accident in 2011. The behaviour of suspended sediment substances, incorporating the effects of deposition and pickup, was assessed using a mixed-sediment particle dynamics model. The concentrations of ^137^Cs adsorbed on fine sediment particles of each size fraction were determined. Removal of ^137^Cs from the cardinal urban river channel had significant effects on both long-term decline, including extreme flash flood events, and the dynamic and time-dependent behaviours of interspersed ^137^Cs and sediment activity. A novel advanced interpolation assessment method was used to examine radionuclide activity in terraced land covered with volcanic ash soil in Tokyo, Japan. The assessment procedure has a time-dependent input function and incorporates the effects of mixed-sediment particle dynamics on this time dependence. The results indicated that sediment and ^137^Cs concentrations could decline more rapidly than observed in the Fukushima and Chernobyl regions. This rate of decrease depended on terraces covered with volcanic ash soil, which incorporated the effects of fine sediment behaviour for particle adsorption. In addition, comparatively large impacts were observed during extreme flash flooding events, which were associated with the land cover of the major urban river catchments in Tokyo. This work provides a new perspective for understanding ^137^Cs behaviour associated with reproduction of sediment deposition and prediction of ^137^Cs concentration in the major urban rivers of Tokyo, incorporating the effects of baseline ^137^Cs behaviour with the impact of sediment particle adsorption in a volcanic ash soil-covered terrace.

## Introduction

Radionuclides, including radioactive ^137^Cs, released from the FDNPP accident following the earthquake and tsunami of 11 March 2011, have risen to critical mass through interspersed deposition on surfaces, mainly through rainfall and discharge into water systems. Radioactive contaminants have accumulated in unusual environments, such as the river channels of urban areas, including within the perimeter of Tokyo (Ministry of Education Culture Sports Science and Technology, 2014; Mouri *et al*., (2014a)^1^; Yamashita *et al*., (2014)^2^; Mouri, (2016a)^3^). Researchers have been investigating and estimating the dynamic behaviour and long-term trends of radionuclides following the 1986 accident in Chernobyl (Vakulovsky *et al*., (1994)^4^; Monte *et al*., (1997)^5^; Smith *et al*., (2000)^6^) and the 2011 accident in Fukushima (Sakaguchi *et al*., (2012)^7^; Nagao *et al*., (2013)^8^; Mouri *et al*., (2014a)^1^). For example, the total levels of ^137^Cs in the grab samples collected in Shinjuku (Tokyo Metropolitan Area) and Onagawa (Miyagi Prefecture) in March 2011 were 8.0 kBq/m^2^ and 10.0 kBq/m^2^, respectively, according to the Tokyo Metropolitan Institute of Public Health and the Environmental Radioactivity Research Institute of Miyagi (MEXT, (2011b)^9^; MEXT, (2012)^10^). A grab sample is defined as an individual volume of water. Grab samples were collected for extreme conditions, while composite samples are composed of a collection of aliquots collected over an extended period of time on either a flow or time basis. The major factors controlling the delivery of ^137^Cs in rivers are affected by land cover types for both urban and rural regions. Pratama *et al*. (2018)^11^ reported a runoff coefficient (*f*) in urban regions of approximately 0.7 and delivery coefficient (*f-* ^137^*Cs*) in rural regions of approximately 0.35 in 2011. The delivery coefficient was defined as the ratio of outflow to deposition of ^137^Cs on the land surface. Tsuruta *et al*. (2017)^12^ reported *f* and *f-* ^137^*Cs* values of approximately 0.5 and approximately 0.2 in forested mountainous regions in 2011. The delivery coefficient (*f-Cs*) in the Chernobyl region was reported to be less than 0.1 (Konoplev *et al*., (2016)^13^). A number of methods have been used for deactivation and rehabilitation of radioactively contaminated soils in Japan to reduce surface soil contamination and delivery of pollutants into the water (Komissarov and Ogura, (2018)^14^; Gupta and Voronina, (2019)^15^; Nakanishi and O’Brien, (2019)^16^). Iitate Village, Fukushima Prefecture, was contaminated with radioactive fallout due to the FDNPP accident on 11 March 2011. Nonetheless, evacuation was cancelled on 31 March 2017, except for areas to which it was difficult to return. Since 1994, Iitate Village, which is located approximately 20 km west of the FDNPP, has been the subject of support practices from the viewpoint of sustainable rural planning. The effects of decontamination measure and ^137^Cs behaviour in that area have been reported (Itonaga, (2019)^17^). For instance, Itonaga (2019)^17^ reported the situations of radiological contamination and attempt of decontamination and indicated that 1) the initial average level of ^137^Cs in the surface layers of both residential and mountainous land was approximately 18000 Bq/kg in 2011. The ^137^Cs in the soil of the urban residential region was addressed by removing soil up to a depth of 30 cm. The level of ^137^Cs in the forest soil surrounding the residential land after decontamination was 3000 Bq/kg. 2) However, the topsoil of the mountainous land has not been sufficiently decontaminated and still has a ^137^Cs level of 17000 Bq/kg. 3) The ^137^Cs in the soil of the mountainous region was addressed by felling of trees and the natural radioisotope decay. 4) Residual ^137^Cs remains in the surface soil of mountainous land near the residential area and a high air dose rate persists despite decontamination measures. 5) Although the surface layers have been decontaminated, ^137^Cs has been adsorbed into fallen leaves within 20 m of the mountain, adjacent to the residential area. 6) The ^137^Cs was identified that the permeation of ^137^Cs as much remains in the surface soil by the dominant plant element particularly leaves, tree species and into the soil of approximately 30% are getting deeper layer as infiltration phenomenon and delivery of pollutants into the river water. 7) There are concerns that ^137^Cs deposited in the environment will migrate to paddy fields through hydrological pathways and cause serious and long-lasting damage to agricultural activities. 8) In addition, the effects of ^137^Cs migration have been examined and the potential bioavailability of ^137^Cs to paddy rice had been quantified by comparing ^137^Cs inventories in paddy field soil with the ^137^Cs entering the paddy fields in irrigation water from three catchments located 40–50 km from the FDNPP (Yoshikawa *et al*., (2014)^18^). 9) Increases in the dissolved ^137^Cs fraction are approximately 3.0%, suggesting that channel water is increasing adsorption of ^137^Cs onto soil particles due to ^137^Cs migration in irrigation water since the Fukushima accident (Itonaga, (2019)^17^). Yoshikawa *et al*. (2014)^18^ studied the influence of ^137^Cs entering paddy fields via irrigation water, and reported ratios of ^137^Cs inflow load and ^137^Cs concentration already in the soil of 0.03–0.05%. This indicates that the effects of ^137^Cs in paddy fields can be significantly decreased via adsorption of ^137^Cs onto soil. In the case of the Tokyo Metropolitan Area, ^137^Cs decontamination occurred immediately due to adsorption by volcanic ash soil during rainfall runoff events as a dominant natural phenomenon. Previous studies have reported initial rapid increases in ^137^Cs concentrations in lake waters as a result of rapid wash-off, a slow decline as a result of soil fixation and redistribution and a very long-term decrease to equilibrium conditions (Imanaka, (2013)^19^). These results were linked to concentrations of suspended sediment (Smith *et al*., (1995)^20^, (2000)^6^; Timmsa *et al*., (2005)^21^); the wash-off of ^137^Cs from forests and farmlands has also been investigated in detail (Bonnett, (1990)^22^; Walling and Quine, (1992)^23^; Poreba *et al*.^24^; Teramage *et al*.^25^; Yoshikawa *et al*.^18^; Yoshimura and Yokokuda, (2014)^26^). By contrast, although data on regional and temporal variation in ambient radioactivity levels in urban areas are available (Mueck and Steger, (1991)^27^; Erlandsson and Isaksson, (2006)^28^; Nuclear Regulation Authority, (2014)^29^), investigations of runoff into urban rivers following both accidents are surprisingly limited.

There have been numerous studies involving both laboratory experiments and theoretical approaches to elucidate the partitioning of ^137^Cs and other radionuclides between the organic and inorganic components of soils, and between the different grain size fractions of mineral soils and sediments (Tamura and Jacobs, (1960)^30^; Coleman *et al*., (1963)^31^; Tamura, (1964)^32^; Lomenick and Tamura, (1965)^33^; Sawhney, (1972)^34^; Francis and Brinkley, (1976)^35^; McHenry and Ritchie, (1977)^36^; Alberts and Muller, (1979)^37^; Evans *et al*., (1983)^38^; Bunzl and Schultz, (1985)^39^; Bachhuber *et al*., (1986)^40^); Livens and Baxter, (1988a)^41^; Livens and Baxter, (1988b)^42^; Maguire *et al*., (1992)^43^; Walling and Woodward, (1992)^44^; Hird *et al*., (1995)^45^). The concentrations of these radionuclides were almost constant across a range of particle size classes (Alberts and Muller, (1979)^37^; Walling and Woodward, (1992)^44^; Livens and Baxter, (1988b)^42^). To understand the distributions of ^137^Cs and other radionuclides between the organic and inorganic fractions of soils and sediments, and between different particle size fractions within mineral components, it is important to consider the initial state of the sample investigated.

The principal goal of investigating the amphibious effects of indigenous particle diameter distributions and the time-dependent behaviours of sediment and ^137^Cs concentrations was to provide insights into the long-term storage of ^137^Cs that would allow most of the radionuclide components to decay to stable products. Olson *et al*. (2002)^46^ used the content of fine suspended particles (such as fly ash), magnetic susceptibility, magnetic minerals, and organic carbon as indicators of soil erosion. The behaviour of radioactive caesium in sediment is largely controlled by adsorption to the surfaces of fine particles and subsequent migration through soil erosion and transfer; such movements can be estimated using technogenic magnetic tracer methods in slightly eroded Chernozem soils (Huffman and Huggins, (1986)^47^; Gennadiyev *et al*., (2005)^48^). Following the Chernobyl accident, a number of studies evaluated the transfer ratio of ^137^Cs from contaminated catchments to rivers (Hilton *et al*., (1993)^49^; Vakulovsky *et al*., (1994)^4^; Monte, (1995)^50^; Smith *et al*., (1995)^20^; Smith *et al*., (1997)^51^; Kudelsky *et al*., (1996)^52^). Temporal changes in concentrations of ^137^Cs have also been studied in river water (Linsley *et al*., (1982)^53^). Previous studies have modelled ^137^Cs concentrations, but it has not been possible to accurately determine the significance and errors in parameter values because of the complexity of the input functions (Linsley *et al*., (1982)^53^; Monte, (1997)^5^). Correspondingly, the changes in concentrations of Chernobyl-derived ^137^Cs in surface waters were linked quantitatively to its declining chemical availability in soils (Smith *et al*., (1999)^54^). Empirically distributed ^137^Cs redistribution models, which employ algorithms for soil loss calculation such as RUSLE, have been applied to uncultivated and cultivated sites in southeast Australia (Martinez *et al*., 2009; Teng *et al*., (2016)^55^). To this end, we modified a novel method for rural areas proposed by Mouri *et al*. (2014a)^1^ to Fukushima-derived ^137^Cs in the urban region of Tokyo, Japan, to compare the mobilities of sediment and ^137^Cs components between selected urban rivers to provide novel insights that can be applied to different urban regions around the world.

The Tokyo Metropolitan Area has been affected by fallout of radioactive substances, particularly caesium- 137 (^137^Cs) released by the Tokyo Electric Power Fukushima Daiichi Nuclear Power Plant (FDNPP) accident caused by the Great East Japan Great Earthquake in March 2011. This study focused on the Tokyo Metropolitan Area, as a strongly affected area with major economic and social importance. The area is an economic centre with a population of 30 million. In the Fukushima region, impacts associated with the reproduction of sediment deposition and prediction of ^137^Cs concentration have been assessed by Mouri *et al*., (2014a)^1^. Therefore, this study is applicable to both the Tokyo Metropolitan Area and Fukushima region, as well as other regions with similar terrain. In addition, a previous study showed that ^137^Cs could be greatly adsorbed to fine sediment particles (Mouri *et al*., (2014a)^1^). The present study assessed the general characteristics of ^137^Cs behaviour, incorporating the effects of sediment adsorption as an important element for understanding the environmental context in the primary urban rivers of Tokyo, and their differences from the Fukushima region. Several important parameters were also identified in cardinal urban rivers in Tokyo, Japan. Sedimentary geochemistry and related phenomena were also incorporated into the assessment; in particular, sediment characteristics have a number of important effects in the context of the environment and climate systems, with both environmental and social impacts (Shiozawa *et al*., (2013)^56^; Grygar *et al*., (2018)^57^; Liu *et al*., (2018)^58^; Apler *et al*., (2019)^59^; Guo *et al*., (2019)^60^; Abdou *et al*., (2019)^61^).

## Methods

### Site and dataset descriptions

**Figure 1:**
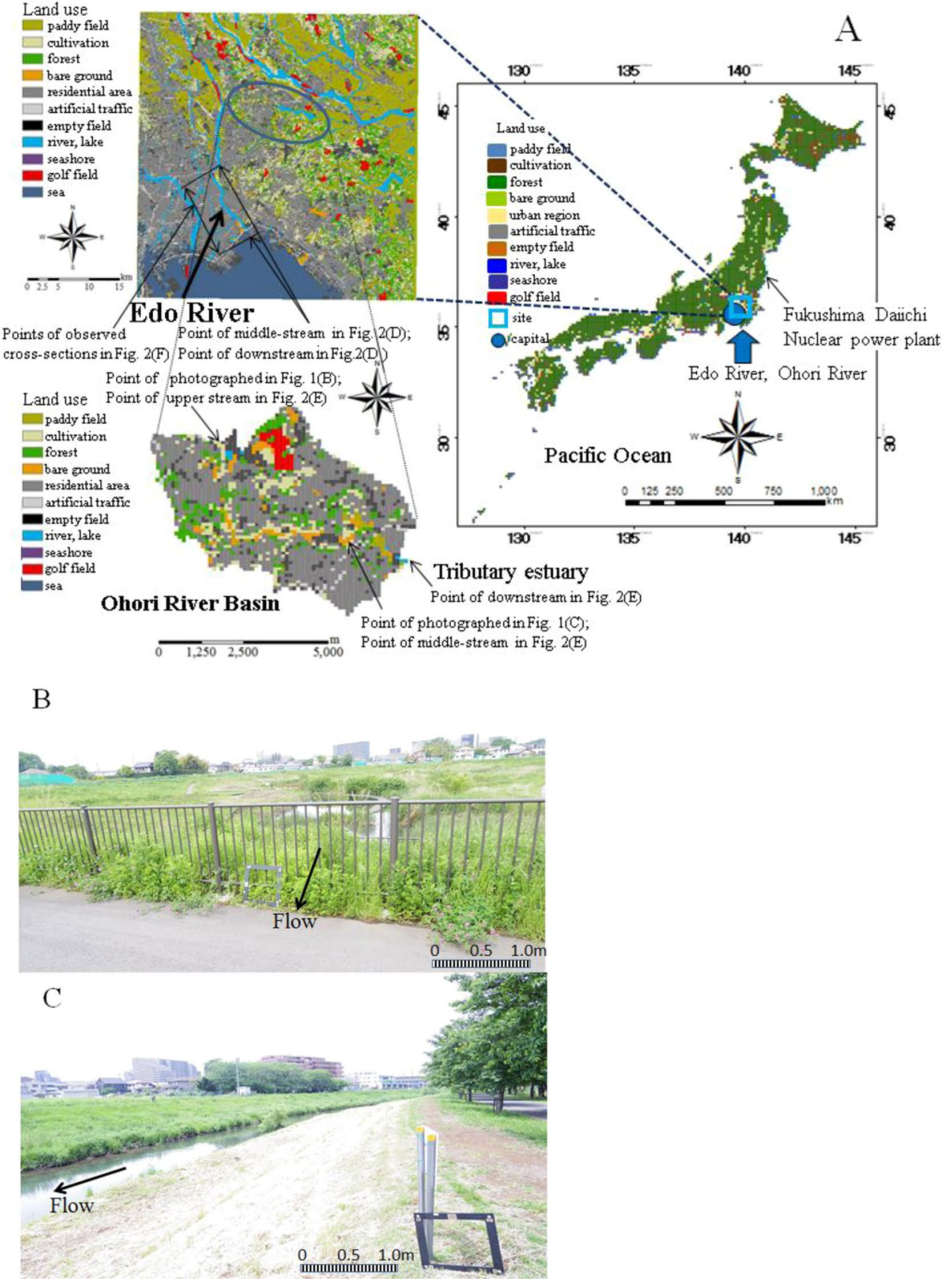
(**A**) The distribution of land use types in the selected typical urban catchment of the Edo Ohori Rivers located at the perimeter of the Tokyo region. The water was supplied to the Ohori River from the Tone River during dry weather. Data are from National Digital Information (DNI). (**B**) Site of the upstream end. (**C**) Site of the midstream region. The photographs in (**B,C**) were obtained from the study field.

**Figure 2:**
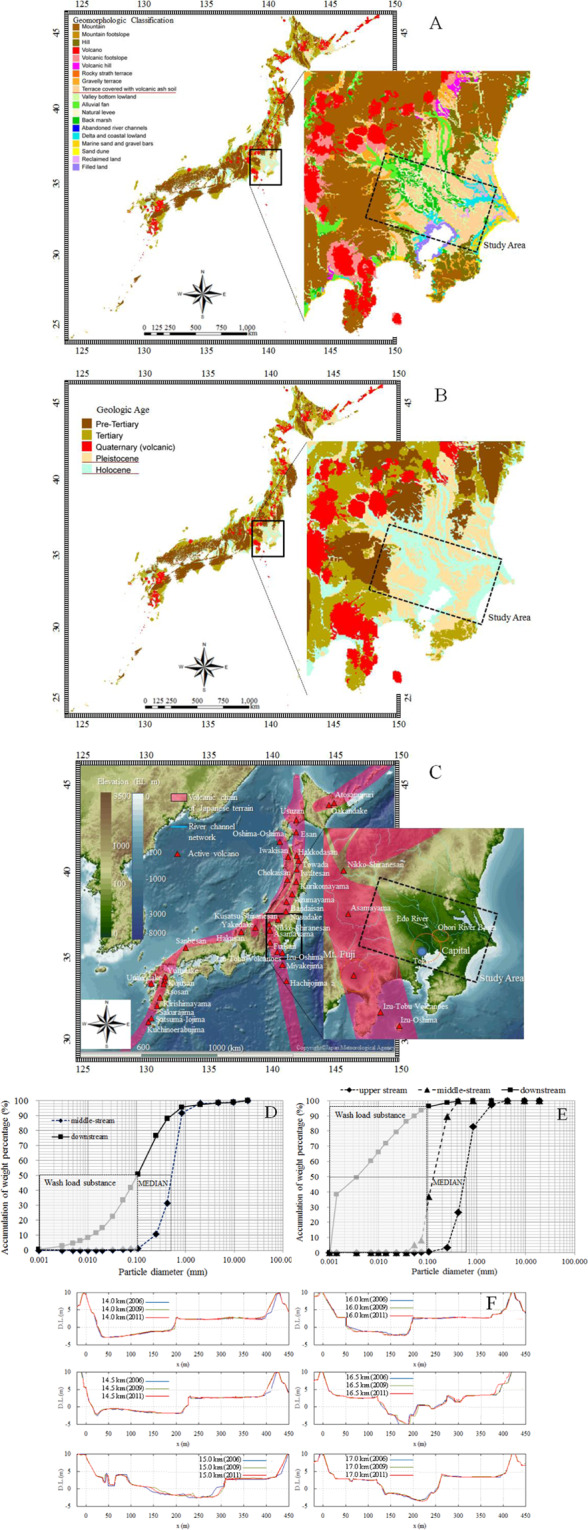
(**A**) Geomorphological context in the Tokyo region. Data were collected from the Digital National Information (DNI). (**B**) Geological age in the Tokyo region. Data were collected from the Ministry of Land, Infrastructure, Transport and Tourism (MLIT) and Chiba Prefecture. (**C**) Volcanic chain of Japanese terrain. Data were collected from DNI and the Japan Meteorological Agency. Data were collected from DNI and the Japan Meteorological Agency. (**D**) Particle size distributions of stream bed material in the Edo River. Data were collected from the MLIT and Chiba Prefecture. (**E**) Particle size distributions of stream bed material in the Ohori River. Data were collected from the MLIT and Chiba Prefecture. (**F**) Spatiotemporal variation in the cross-section in the Edo River. Data were collected from the MLIT.

Numerical analyses for current and long-term predictions were conducted for two typical urban rivers in the Tokyo region, the Edo River and the Ohori River, which have sporadic high ambient concentrations of radioactive substances in soil, particularly ^137^Cs at approximately 5,000 Bq/kg (MEXT, (2012)^10^). The Edo River is a tributary of the Tone River. The catchment area of the Edo River Basin is 200 km^2^, located on the eastern border of the Kanto plane. The river course is approximately 32.5 km from the estuary barrage of the Edo River, and was separated into 53 sections (at ~500 m intervals) to evaluate the spatial variability of sediment (MLIT, (2014)^62^). The highest altitude in the basin is at the upper end of the distributary, with an elevation (EL) of 8.6 m based on Tokyo Peil (TP) as the mean sea level of Tokyo Bay of Japan. The dominant surface geological feature is a loamy layer over Holocene sediments of the Kanto Plain, covered by a residential area (approximately 48%). The average annual rainfall is approximately 1,279 mm/yr. About 98% of the population (3,350,000 of 3,400,000 people) is served by the local sewer system. A drinking water treatment plant is located on the main river, 14.0 km from the estuary barrage. The Ohori River is approximately 5.62 km from the estuary barrage, with 121 river cross-sections (at ~50 m intervals). There is an artificial lake (area ~4 km^2^) at the main river estuary, which is used for agriculture and fisheries activities in this region. The annual river water flow is approximately 100 m^3^/s during non-flood periods, although a flow of ~3,700 m^3^/s (for 1,959 s) was recorded in a flood period (MEXT, (2011a)^63^). The Ohori River is located in Kashiwa City, Chiba prefecture. The catchment area is 31 km^2^ and the average river discharge downstream is 1 m^3^/s. The land use is predominantly residential (housing land, 57.5%; road, 2.0%), unlike that in the Fukushima prefecture, which is 71% forest (Forestry Agency, (2014)^64^). During dry weather, 0.5 m^3^/s water is supplied to the Ohori River from the Tone River. The river’s abundant water volume is used to irrigate an area of 6.0 km^2^, mainly from small tributaries and waterways. Mountainous, urban and cultivated areas account for 15.0%, 63.0% and 10.0% of the land area, respectively (Fig. 1A). The upstream end and middle stream are surrounded by residential areas where there are several sources of road ash sediment (Fig. 1B,C). The land use map was generated using Digital National Information. General features are described associated with the Tone River Basin 1A) and the artificial lake, Teganuma Basin (Murakami *et al*., (2015)^65^). Time series of measurements of ^137^Cs in the Edo River in the contaminated Tokyo region were taken from previous reports (MEXT, (2011a)^63^; MEXT (2014)^66^) and temporal variation in the ^137^Cs concentration in soil has been reported by the reports. The time period corresponded to the start of the second phase of the survey of Timms *et al*. (2005)^21^. In addition, grab and composite samples were collected for ^137^Cs every 3 to 4 weeks from May 2012 to January 2015 (Murakami *et al*., (2015)^65^). Grab samples were also collected during wet weather. Composite samples were collected with a suspended sediment (SS) sampler designed to collect time-integrated SS samples (Phillips *et al*., (2000)^67^). Collection ratios are higher at higher flow ratios (Yamashita *et al*., (2014)^2^), such that the sampler preferentially stores SS samples collected during wet weather. Although deposition was spatially variable, it can be assumed that the relative fallout varied little on an annual basis. We measured the actual sediment deposition and the accumulated sediment deposition record in the Tokyo region for the model of the initial conditions and its validation (Yamashita *et al*., (2014)^2^). The water samples were dried to determine the amounts of suspended sediments followed by calculation of ^137^Cs or measurement of turbidity. Grab samples and composite samples were collected beginning in May 2012. In the second stage of this survey (April 2013 to January 2015), along with the collection of grab samples during dry weather conditions and composite samples, time-series grab samples were collected during wet weather (up to 8 samples per event) with an automatic sampler (Teledyne-Isco, USA). In total, 44 dry-weather grab samples, 122 wet-weather grab samples (23 events), and 39 composite samples were collected from May 2012 to January 2015. The water depth and electrical conductivity (EC) were monitored on site with a 750 Area Velocity Flow Module (Teledyne-Isco) and a water quality multiprobe (Data Sonde 5, Hydrolab, USA), respectively. Samples were filtered through prebaked glass fibre filters (GF/F, 0.7 mm pore size; Whatman, UK) for consistency with previous research (Yamashita *et al*., (2014)^2^). The SS concentration was determined from the filtrate volume and the difference between dried filter weights before and after filtration. Filtrates were further filtered through membrane filters (0.2 μm; Advantec, Japan) to obtain the dissolved phase, in accordance with previous research (Murakami *et al*., (2015)^65^). Concentrations of ^137^Cs were analysed on both an SS weight basis (Bq kg·SS^–1^) and a liquid volume basis (Bq/L) for the particulate phase, and on a volume basis for the dissolved phase. The radioactivity was decay-corrected to the collection date. All water and soil samples (dried and sieved to <2 mm), as well as standard reference materials and laboratory standards prepared from standard solutions, were analysed for gamma-ray emissions at energies of 662 keV (^137^Cs) using a high-purity n-type germanium coaxial gamma-ray detector (EGC25–195-R; Canberra-Eurisys, Meriden, CT, USA) with an amplifier (PSC822; Canberra, Meriden, CT, USA) and a multichannel analyser (DSA1000; Canberra). The river channel was a movable bed covered with sediment material, and had the same particle size distribution as riverbed material from the upper end of the stream. The total lengths of the Ohori and Edo Rivers are approximately 5.62 km and 32.5 km, respectively. The sediment material was supplied from approximately 200 m length of balancing reservoir from the upper end of the stream, as shown in Fig. 1B. The average riverbed gradients are from 1.3‰ to 13‰ and river widths are from 50 to 200 m, indicating stable artificial river geomorphology for the Edo and Ohori Rivers. Figure 2A shows the geomorphological context in the Tokyo region where there is terraced land covered with volcanic ash soil of fine particle sediment. Figure 2B shows the geological context in the Tokyo region consisting of Pleistocene and Holocene sediment. Figure 2C indicates the volcanic chain of the Japanese terrain including Mt. Fuji, which is located close to the western edge of the Tokyo Metropolitan Area. Figure 2D,E show the vertical changes in size distributions of surface particles of the terraced land covered with volcanic ash soil. The mixed grain sizes used in the model were based on the results of this investigation and ranged from 0.001 to 100 mm (Ministry of Land Infrastructure, Transport and Tourism [MLIT], (2014)^62^). Grain sizes were divided into 10 representative categories. The D_50_ grain sizes for both rivers consisted of medium sand, fine sand and a silt component from 0.0067 mm (downstream) to 0.43 mm (upstream) in the Edo River, and from 0.10 mm to 0.56 mm in the Ohori River (Table 1). We assumed that wash load (d ≤ 0.2 mm) made almost no contribution to two typical rivers sedimentation and artificial dam site section and it was thus discarded from the analyses, while soil samples of 10 representative categories contributed to ^137^Cs adsorption and therefore the indigenous particle distributions were incorporated from into the analyses (Table 1). The soil constituent percentages indicated that medium (d ≤ 0.63 mm) grain-size sand, fine sand (d ≤ 0.2 mm), silt (d ≤ 0.063 mm), and clay (d ≤ 0.002 mm) were associated with the wash load component, which appeared to incorporate the backwater effect in the downstream sections of both the Edo and the Ohori rivers (Table 2). The sediment particle characteristics indicated that the limited riverbed grain sizes in the river estuaries resulted from the backwater effect. The vertical changes demonstrated that the reservoir controlled sediment deposition and discharge through the backwater effect. Figure 2F shows cross-sectional variation in the river bed in 2006, 2009, and 2011.

**Table 1:** Fundamental sediment characteristics and particle size distribution of the riverbed sediment for Edo River and Ohori River.

Edo River	sediment characteristics		
	upper stream (33.0 km from the river mouth)	middle-stream (15.5 km from the river mouth)	downstream (0.0 km from the river mouth)
maximum grain size (mm)	9.500	2.000	0.850
D_60_ grain size (mm)	0.5405	0.3107	0.0067
D_50_ grain size (mm)	0.4351	0.2841	0.0036
D_30_ grain size (mm)	0.2774	0.2295	—
D_10_ grain size (mm)	0.1384	0.1292	—
uniformity coefficient	3.91	2.4	—
curvature coefficient	1.03	1.31	2.58
**Ohori River**	**sediment characteristics**		
	**upper stream**	**upper stream (4.0 km from the river mouth)**	**downstream (0.0 km from the river mouth)**
maximum grain size (mm)	—	19.000	19.000
D_60_ grain size (mm)	—	0.625	0.158
D_50_ grain size (mm)	—	0.555	0.104
D_30_ grain size (mm)	—	0.411	0.046
D_10_ grain size (mm)	—	0.237	0.012
uniformity coefficient	—	2.63	12.80
curvature coefficient	—	1.14	1.10

**Table 2:** Soil constituent percentages in riverbed sediment of the Edo River and Ohori River.

Edo River	soil constituents percentage (%)				
	pebble	granule	coarse sand	medium sand	fine sand
location	(d ≤ 64.0 mm)	(d ≤ 4.0 mm)	(d ≤ 2.0 mm)	(d ≤ 0.63 mm)	(d ≤ 0.2 mm)
upper stream	0.0	0.0	3.0	70.0	27.0
middle-stream	0.0	0.0	1.0	72.0	26.0
downstream	0.0	0.0	0.0	0.0	43.0
**Ohori River**	**soil constituents percentage (%)**				
	**pebble**	**granule**	**coarse sand**	**medium sand**	**fine sand**
location	(d ≤ 64.0 mm)	(d ≤ 4.0 mm)	(d ≤ 2.0 mm)	(d ≤ 0.63 mm)	(d ≤ 0.2 mm)
middle-stream	1.5	0.4	6.3	81.0	10.0
downstream	1.5	1.2	1.7	19.1	34.5

### Model descriptions

Detailed studies are required to determine the complex behaviours and interactions of radionuclides (Mouri *et al*., (2013b)^68^; Mouri *et al*., (2015b)^69^). In this study, variation in the dynamic behaviour of sediment and ^137^Cs fluxes, including the amphibious effects of sediment particle distribution and the adsorption of ^137^Cs onto individual particle fractions, were assessed following the work of He and Walling (1996)^70^, Smith *et al*. (1999)^54^, (2000)^6^, Viparelli *et al*. (2013)^71^ and Mouri *et al*. (2014a)^1^ based on the catchment simulator modelling framework (Mouri and Oki, (2010)^72^; Mouri *et al*., (2011a)^73^; Mouri *et al*., (2013a)^74^; Mouri *et al*., (2014b)^75^). The catchment simulator can be used to link the terrestrial and aquatic components of catchments in terms of sediment generation and transfer. The catchment simulator was originally developed by Mouri and Oki (2010)^72^. Further description of the simulation process and equations can be found elsewhere (Mouri *et al*., (2011a)^73^; Mouri *et al*., (2013a)^74^; Mouri *et al*., (2014a)^1^; Mouri, (2015a)^76^). The components considered in the model are rainfall runoff, the effects of forest transition features across Japan, and the impact of changing climate. All water fluxes onto the ground surface were aggregated, and a portion of this water, depending on land cover, soil conditions and physical soil properties, became overland flow. Base flow was calculated using the simplified TOPMODEL scheme (Beven, 1997; Beven and Freer, 2001). The model was improved to incorporate the effects of ^137^Cs adsorption onto individual particle fractions and its time-dependent behaviour, following previous research studies (Bunzl and Schultz, (1985)^39^; Alberts and Muller, (1979)^37^; Monte, (1995)^50^; Smith *et al*., (1995)^20^, (1997)^51^, (1999)^54^, (2000)^6^; Hakanson, (2005)^77^; Mouri *et al*., (2014a)^1^. The variability in monthly precipitation in autumn associated with changing climate was projected using simulations of two general circulation models (GCMs), the Model for Interdisciplinary Research on Climate (MIROC) and the Meteorological Research Institute Atmospheric GCM (MRI-GCM). The CO_2_ emissions scenario used in this study was the Representative Concentration Pathway (RCP) scenario and the climate models were MIROC3.2 and MRI-CGCM 2.3.2, based on the climate predictions of CMIP5. Dynamic and statistical downscaling methods were applied to the four GCMs (MIROC, MRI-GCM, HadGEM, and GFDL) under the RCP scenario to bridge the gap between available GCM outputs and the climate inputs required for impact models (Mouri, (2016b)^78^). Estimation of spatial and temporal variation in monthly precipitation incorporating the effects of the forest transition under the MIROC, MRI-GCM, HadGEM, and GFDL scenarios was carried out using current and future forest variation, and then applied for 50-year forecast projection to the period of 2041 to 2050. This method results in comprehensive prediction of the variability in monthly precipitation data associated with changing climate, which is used as the boundary condition in the sediment and ^137^Cs numerical dynamic models of the catchment simulator. Although the resolution of the GCM is approximately 1 km, the catchment simulator predicts the effects of flooding, water level variation and other hydrological phenomena with high precision. The spatial resolution of the catchment simulator is 50 m and its temporal resolution is less than 1 s. GCM ensemble prediction could be applied to provide boundary conditions for the catchment simulator as a regional high-precision model. The ratio of transfer of radioactivity from catchments to surface waters was quantified using the runoff ratio, defined as the concentration ratio of radioactivity in the river (Bq/m^3^) to the mean radioactivity deposition in the catchment (Bq/m^2^). The ratio of runoff may be modified using relatively simple exponential functions that simulate declining radioactivity concentrations in discharge over time (Monte, (1995)^50^; Smith *et al*., (1997)^51^). In addition, the runoff of radionuclides to surface waters was characterised by the fast flushing of recently deposited radioactivity, followed by slower transfers during subsequent years. It has been shown that ^137^Cs concentrations in river channels initially declined after fallout (Monte, (1995)^50^; Smith *et al*., (2000)^6^). For longer timescales, ratio of runoff were assumed to decline much more slowly as immobilisation and redistribution processes tended towards a steady state. The concentration of the radionuclide in runoff water *C*_*t*_ (Bq/m^3^) resulting from a spike deposition has been applied. The parameters represent an exponential decline in runoff over the first few years following deposition, as a result of the fixation processes of radionuclides in catchment soils (Smith *et al*., (1999)^54^).

The function should be viewed as a general expression for the description of radionuclide mobility in catchments. The equation shown is for a special case where, fallout is assumed to be equal to zero in all except one year; this special case was used for Tokyo data as all fallout was assumed to have occurred in 1986, the year of the Chernobyl accident. For a time-dependent input function, with fallout occurring over several years due to radionuclides in runoff water in the year after the beginning of fallout, *C*_*t*_(*j*) (Bq/m^3^) is given by:

1 $${C}_{t}(j)=AD(j)+\mathop{\sum }\limits_{i=1}^{i=j}D(i)(B{e}^{-(\lambda +{k}_{1})(j-i)}+C{e}^{-(\lambda +{k}_{2})(j-i)}),$$

where *A* (m^–1^), *B* (m^–1^) and *C* (m^–1^) are empirical coefficients that represent the fast flushing of activity, the slow decline as a result of soil fixation processes and the long-term runoff fraction, respectively, and *λ* is the decay constant of the radionuclide, which is 0.023 y^–1^ for ^137^Cs. *D*(*j*) (Bq/m^2^) is the deposition level during 1 year. *AD(j)* (Bq/m^2^) is the initial radioactivity concentration in rainwater. The *A* coefficient has a positive value during the first year following deposition and is zero thereafter. The parameter *k*_1_ represents an exponential decline in runoff over the first few years following deposition as a result of the fixation processes of radionuclides in catchment soils (Smith *et al*., (1999)^54^). The parameter *k*_2_ represents a longer term decline over a period of decades. To identify the other parameters, the empirical coefficients *A*, *B* (m^−1^), *k*_2_, and *e* were adopted from Smith *et al*. (1999)^54^. In addition, calibration was conducted using the iterative method for *C* and *k*_1_, and the resulting values were applied to *C* (0.28 m^−1^), *k*_1_ (0.035), and *D(i)* (5.0 kBq/m^2^) (MEXT, (2014)^66^). The coefficient *D(i)* was calculated to incorporate the effects of the natural decay coefficient of ^137^Cs associated with the coefficient *D(j)*, which was applied during the prediction phase. Equation (1) and its special cases were fitted using a nonlinear least-squares regression procedure with a derivative-free algorithm (Rios and Sahinidis, (2013)^79^). The adsorptions of ^137^Cs onto different particle size fractions of soil material, based on the relationship between specific surface area for individual particle fractions and ^137^Cs concentrations, were obtained using the method of He and Walling (1996)^70^, which incorporates the effects of the concentrations adsorbed onto soil particles of specific surface areas, as shown in Eq. (2). The power function *S*_*sp*_ was used to represent the relationship between ^137^Cs concentrations *C*_*n*_(*S*_*sp*_) and the mean specific areas *S*_*sp*_ of the fractions. The value used, 22.1, is the empirical coefficient associated with the results of a previous experiment (He and Walling, (1996)^70^). The concentrations adsorbed onto soil particles of specific surface areas can be expressed as:

2 $${C}_{n}({S}_{sp})=22.1{S}_{sp}^{0.60},$$

The particle size effect was incorporated into the dynamic numerical model associated with river sediment and ^137^Cs behaviour. Sediment and ^137^Cs behaviour was predicted using a dynamic numerical model incorporating the effects of the sediment particle distribution. The numerical dynamic model within the catchment simulator framework predicts geomorphological variation, the volume of particles of each diameter, sediment concentration, ^137^Cs concentration, and other factors (Mouri *et al*., (2014a)^1^). The adsorption levels of ^137^Cs onto particles of different size fractions in soil, based on the relationship between the specific surface area of an individual particle fraction and ^137^Cs concentration, were obtained using the method of He and Walling (1996)^70^, which incorporates the effects of the concentration adsorbed onto soil particles of specific surface areas described in Eq. (2) into a conversion of Eq. (1). The enrichment ratio for ^137^Cs associated with sediment eroded from cultivated soil can also be defined from Eq. (2) using the known radionuclide activities and mean specific surface areas of the samples. In addition, the wash load, which is the dominant process for ^137^Cs adsorption, was obtained following the work of Egiazaroff (1965)^80^, Ashida and Michiue (1972)^81^ and Mouri *et al*. (2014a)^1^.

### Other analyses conditions

The main purpose of this analysis was the functional verification of the actual environmental and social contexts, based on the MLIT dataset (MLIT, (2014)^62^) that considered sediment and its particle distribution and the ^137^Cs adsorption onto individual particle fractions in selected typical urban rivers in a contaminated region of Tokyo. Thus, the correction coefficients of the bedload and suspended-type formulas were in agreement with the results of observational records in terms of flood plain, riverbed variation and ^137^Cs concentration (MLIT, (2014)^62^). The observed river data were provided by the MLIT (2014)^62^. The sediment yield was determined as a boundary condition at the upstream regions of the selected rivers, removing the need for a catchment simulator framework (Mouri *et al*., (2011a)^73^, (2011b)^82^). These systems were applied as the boundary conditions for calculations in this selected region, and the time-series of river flows, sediment behaviour and the effects of amphibious sediment particle adsorption and the impact of time-dependent behaviour in typical urban rivers in Tokyo, Japan, for ecotoxicology and environmental safety assessment.

## Results

### Vertical sections of sedimentation

**Figure 3:**
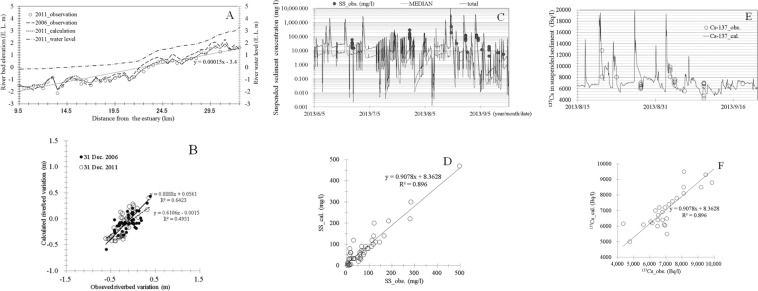
(**A**) Vertical sections of the validation calculations in terms of cross-sectional variation in the Edo River. Data were collected from the Ministry of Land, Infrastructure, Transport and Tourism (MLIT) and Chiba Prefecture. (**B**) Scatter plot of validation data against observed riverbed variation in the selected rainy season, including wet and dry conditions. Data were collected from the MLIT.

Figure 3A shows the vertical sections of sedimentation. The simulated results for riverbed variation were validated using observational data for the Edo River throughout cross-sections, and showed good reproducibility (Fig. 3A). Overall, the results of the model were in agreement with the observed erosional/depositional processes for the Edo River. Downstream, neither river showed any large differences in sediment deposition caused by the 3 years of river flow, including both flood and non-flood periods, but differences were evident upstream due to the backwater effect. Comparatively little sediment formed downstream, which lacked an estuary barrage. Although the degree of change differed locally, the calculated results generally matched the values observed by MLIT (Fig. 3B). The vertical sections of sedimentation were validated, incorporating the effects of sediment particle dynamics, allowing vertical changes in the particle size distributions to be predicted with high precision. Thus, the results of this study can be applied to prediction of ^137^Cs concentrations, incorporating the effects of sediment particle adsorption.

### Vertical changes in particle size distributions

**Figure 4:**
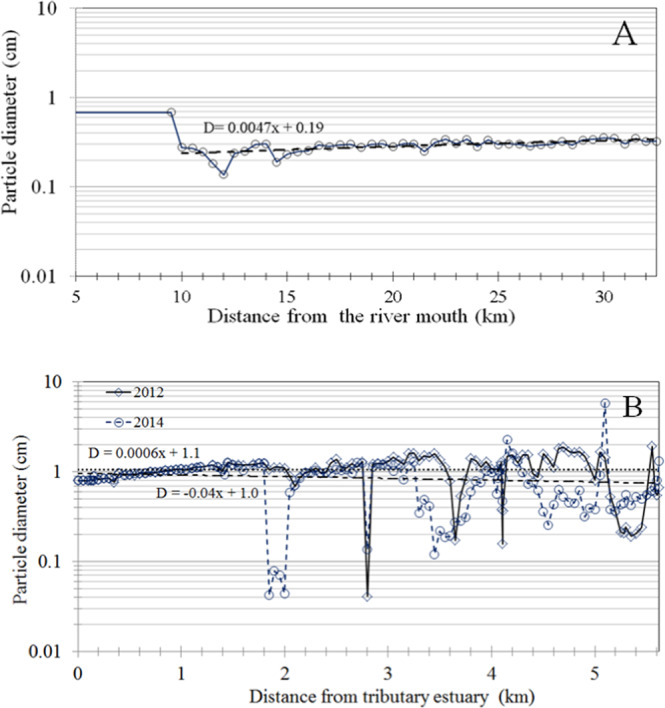
(**A**) Estimated vertical profile of particle diameters, incorporating the effects of mixed particle diameter distributions, in the Edo River. (**B**) Estimated vertical profile of particle diameters, incorporating the effects of mixed particle diameter distributions, in the Ohori River.

Figure 4 shows vertical changes in the median size distributions of sediment particles accumulated on the riverbed surface, incorporating effects of the sediment particle distributions in the Edo River and the Ohori River. The mixed soil particles assessed in the simulation were based on the results of a previous investigation, and ranged from 0.001 to 100 mm (MEXT, (2014)^66^). Soil sample particles were divided into 10 representative categories. We assumed that wash load (d ≤ 0.2 mm) made almost no contribution to sedimentation in two typical rivers and artificial dam site section, and it was therefore discarded from the analyses, while soil samples of 10 representative categories contributed to ^137^Cs adsorption. The results showed the limited main channel grain sizes resulting from the backwater effect along the estuary barrage segment. Thus, the vertical changes indicated that the estuary barrage segment controlled sediment deposition and discharge through the backwater effect.

### Time-dependent behaviour and short-term variation in ^137^Cs concentration and sedimentation

**Figure 5:**
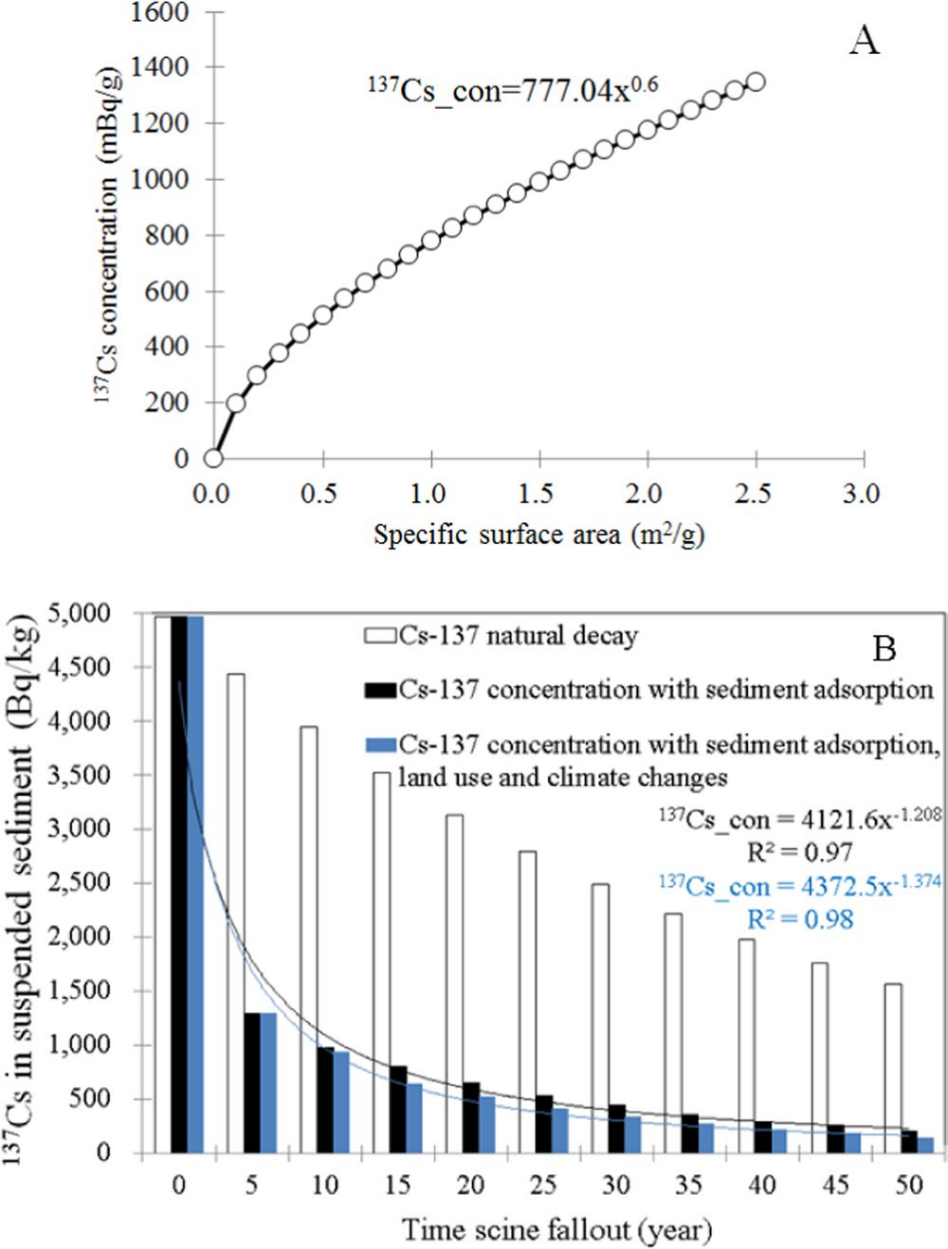
(**A**) Correlation between ^137^Cs concentration and specific surface area. (**B**) Time response of ^137^Cs spillage concentrations to various catchment environmental factors in the urban catchment of the Edo River basin, following the work of Mouri *et al*. (2013).

On very long timescales, small amounts of some of the longer-lived radionuclides can be transported back to the biosphere, typically by migration on the land surface. Therefore, it is necessary to determine the long-term radiological impacts, incorporating the effects of long-term declines in ^137^Cs concentrations that are likely to occur (Penttilä *et al*., 1993; Sansone *et al*., 1997; Thorne, 1998; Smith *et al*., (2000)^6^; Mouri *et al*., (2014a)^1^. Parameter values obtained from the post-Chernobyl accident measurements (Smith *et al*., (1999)^54^, 2000) were applied to the model for post-Fukushima estimations. The correlation between ^137^Cs concentration and specific surface area was determined for prediction of sediment and ^137^Cs behaviours in the Edo and Ohori rivers, incorporating the dynamic numerical sediment and ^137^Cs model from the catchment simulator (Fig. 5A). The coefficient of the exponential function from the works of Yamashita *et al*. (2014)^2^ and Murakami *et al*. (2015)^65^ was applied. The Chernobyl accident measurements of the time-dependent input function and parameter values in Eq. (1) were used, which incorporate the effects of the concentrations adsorbed onto soil particles of specific surface areas expressed in Eq. (2). The ^137^Cs concentrations in the Edo river catchment for the 50-year post-Fukushima period, 2011–2061, were assessed (Fig. 5B). The initial fallout input was obtained from the MEXT observation records (MLIT, (2014)^62^). Five years after the Fukushima accident, the estimated ratio of decline in ^137^Cs concentrations were higher than those predicted by redistribution processes in the Chernobyl region. The decline in ^137^Cs concentrations during this period is attributed to the fast mobilisation of both water and ^137^Cs with incorporation of the amphibious effects of sediment particle adsorption over urban land surfaces and river flow velocity, based on recent post-Fukushima measurements (Murakami *et al*., (2015)^65^). The involvement of the fast mobilisation of both water and ^137^Cs, and the effects on their decay curve contexts, exemplified the amphibious effects of sediment particle adsorption during wet periods known as the flash flood phenomenon. As their decay curve contexts for the selected region caused them to devote geomorphological and hydrological context associated with typical characteristics urban region, both water and ^137^Cs transported fast and accurate. Slow adsorption reactions are expected to reach equilibrium and the ratio of decline in ^137^Cs concentrations in runoff water will decrease, trending towards a faster ratio of decline that would result from physical decay and redistribution processes in the urban region. Some groups have predicted increases in erosion over the coming decades due to climate change (Li and Fang, (2016)^83^). In this study, the calculations incorporated the effects of climate change by incorporation of general circulation model (GCM) climate change scenarios with 50-year runoff forecasts (Mouri, (2016b)^78^). Four GCMs were selected from the Coupled Model Intercomparison Project Phase 5 (CMIP5) ensemble to provide the driving factors: the Model for Interdisciplinary Research on Climate (MIROC), the Meteorological Research Institute Atmospheric General Circulation Model (MRI-GCM), the Hadley Centre Global Environment Model (HadGEM), and the Geophysical Fluid Dynamics Laboratory (GFDL) climate model. The decreasing concentration during this period is attributed to the rapid mobilisation of both water and ^137^Cs with incorporation of the amphibious effects of forest transition and runoff, based on GCM climate change scenarios. The correspondence of the projected forest type and typical land cover was estimated from the Forestry and Forest Products Research Institute (FFPRI) dataset (Mouri, (2016b)^78^). The ratio for the present conditions associated with land cover and climate revealed an increase in the average runoff in the Tokyo Metropolitan Area of approximately 21%. This result could indicate the typical characteristics of major urban catchments, which depend mainly on sediment and ^137^Cs interactions with asphalt and artificially constructed surfaces as well as the characteristics of modified urban rivers that move faster than those in rural regions. Because these typical conditions cause fast water movement and flash flooding events, increasing discharge over short (daily to yearly) time scales, ^137^Cs in suspended sediment in the catchment could be reduced for long time scales (up to decadal). Based on the time-dependent behaviour of ^137^Cs, the short-term variation in ^137^Cs concentrations were simulated using the catchment simulator framework (e.g., Mouri *et al*., (2014a)^1^). Overall, the time-series variation was able to reproduce the observed MLIT records incorporating both digital and survey high-resolution data sets with accuracy of approximately 10–50 m. The sediment and ^137^Cs concentrations during short term flooding events showed good agreement with the MLIT data (Fig. 3C–F). Calculated values were generally in agreement with experimentally observed sedimentation rates and ^137^Cs concentrations (MLIT, (2014)^62^; Murakami *et al*., (2015)^65^). Vertical sections showed not only the observed sedimentation form but also the sediment flux (MLIT, (2014)^62^). A comparison of calculated and experimental streambed changes confirmed the validity of the calculated results, although local differences in factors such as delta formation were observed. Observed vertical changes in the particle size distribution validated the model’s classification of the effects of continuous river structures on sediment movement (MEXT, (2014)^66^). From the results of these calculations, grain sizes were reduced at dam sites due to the presence of a reservoir. The calculated ^137^Cs concentrations were in agreement with experimentally observed erosional and depositional tendencies (Murakami *et al*., (2015)^65^), with sediment behaviour similar to that in an artificial river. The agreement between calculated trends and experimental observations confirmed that the model correctly characterised the delivery of water, sediment, and ^137^Cs in an artificial river system. The agreement of sedimentation form data and calculated incoming and outgoing fluxes with experimental results also supports the validity of the model. SS and dissolved ^137^Cs decreased over time and with decreasing water depth (relative standard deviation (RSD) during the event was 47% for SS and 33% for dissolved ^137^Cs), whereas particulate ^137^Cs varied little (RSD, 9%). The particulate distribution of ^137^Cs increased with increasing SS, ranging from 10% to 58% during dry weather and 59% to 93% during wet weather. The dominant distribution of ^137^Cs during wet weather is in accordance with findings from Fukushima. The negative correlation of partition coefficient (apparent Kd) with SS during wet weather could be explained by the increase in particle size corresponding with increasing SS during extreme flash flooding events (Mouri *et al*., (2014a)^1^). Without correction for surface area, the relationship between dissolved ^137^Cs on a liquid volume basis and particulate ^137^Cs on an SS weight basis was weak; with correction, this relationship strengthened. Therefore, the difference in specific surface area of sediment particles during events contributed to the variation in the apparent Kd of ^137^Cs during wet weather. In addition, this study indicates that the effects of the sediment particle distribution and SS concentration are useful for predicting sediment and ^137^Cs behaviour in the river channel during wet weather, and that the specific surface area plays an important role in driving these behaviours. This result could be related to the characteristics of land cover in the urban area as well as those of the artificial urban river.

Further studies are necessary to identify the factors causing variation in the partition coefficient (apparent Kd) to support high-precision prediction. Nevertheless, this study shows that the effects of the sediment particle distribution and SS concentrations are useful for predicting sediment and ^137^Cs behaviours in a river channel during wet weather, and that specific surface area plays an important role in their variation. Figures 1 to 4 provide valuable information about the typical influences of the sediment particle distribution of volcanic ash soil, urban land cover and artificial river characteristics. In addition, this study provides useful insights into the differences between the Fukushima region (rural) and Tokyo region (urban), building upon previous works that have focused on the Fukushima region. Information gained from the Fukushima region has been included in this manuscript.

**Figure 6:**
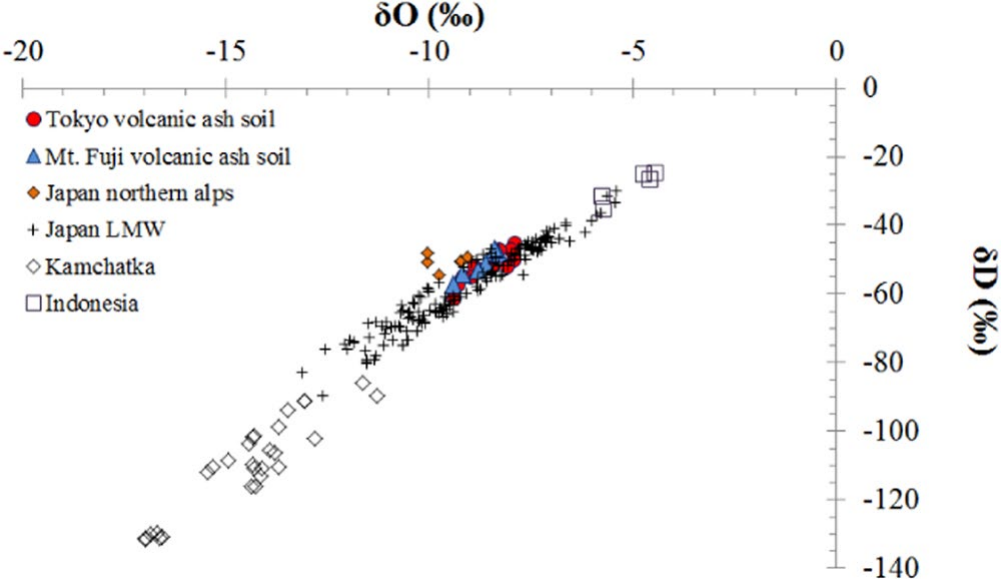
Plot of dD vs. d^18^O values in the terraced land covered with volcanic ash soil samples (TCVAS) from eastern Tokyo. Stable isotopic compositions varied as a function of the volcanic ash soil in the cardinal urban river sediment. Red circles: oxygen and hydrogen isotopes in the TCVAS. Blue circles: oxygen and hydrogen isotopes in the volcanic ash soil of Mt. Fuji. Black rhombuses: Kamchatka local metric water in 2012 and 2013 with additional data added from Mouri *et al*. (2014). Black squares: Indonesia local metric water. Black crosses: local meteoric water (LMW) with additional data added from Mouri *et al*. (2011).

Isotopes are variants of a particular chemical element with the same number of protons (i.e., atomic number), but different numbers of neutrons. Figure 6 shows a plot of δD versus δ^18^O, with samples from terraced land covered with volcanic ash soil samples (TCVAS) in the Tokyo region plotted close to the Japanese local meteoric water line (LMW). The Mt. Fuji volcanic ash soil samples showed a similar tendency to the TCVAS isotopes of the cardinal urban river in the Tokyo region, and were slightly off compared to the LMW (Mouri *et al*., (2011b)^82^). Mt. Fuji is located in the Southern Japan Alps close to the Tokyo Metropolitan Area. The line along which samples from TCVAS fall was defined (*n* = 11; r^2^ = 0.79), and was moderately higher than that of the LMW (6.98). The source of the measured water could be TCVAS from the major urban rivers in the Tokyo region and its surroundings, as the samples had similar geomorphological geological age contexts to TCVAS from Mt. Fuji (slope: 7.2), where water percolates through TCVAS. Through sublimation, water enriched with heavier isotopes can be removed from materials in the soil layer. This process would not affect the isotopic composition of the remaining terraced land covered with volcanic ash soil in Tokyo, in contrast to the situation in an evaporating water body where the effects of surface fractionation propagate into the residual liquid through mixing (Mouri, (2016a)^3^). These samples appear to have undergone varying degrees of evaporation and infiltration, as the soil layer is similar to that of Mt. Fuji. Terraced land covered with volcanic ash soil was enriched in moderately heavy isotopic compositions due to evaporation and infiltration of water.

## Discussion and Conclusions

The study was performed to gain insight into novel aspects of sedimentary geology, including the effects of the behaviour of river sediment and radionuclides through the accident of FDNPP in terraced land covered with volcanic ash soil area close to the Tokyo Metropolitan Area.

We assessed the characteristics of terraced land covered with volcanic ash soil, incorporating the amphibious effects of sediment particle size distributions and their time-dependent behaviours, to examine the effects of human activity and a major developed urban area on sediment dynamics and ^137^Cs behaviour in the riverine environment. The river geomorphology and river water record, incorporating the effects of flood events, were also included to identify ^137^Cs fluxes and sedimentation forms. The agreement of sedimentation form with observations showed that the model approach was valid when the detailed effects of sediment particle distributions and the time-dependent behaviour of ^137^Cs were identified. The assessment was also able to reproduce the complex phenomena that occurred in the river in the geomorphological context of terraced land covered with volcanic ash soil. Vertical sections of sedimentation form were in general agreement with the experimental results. Furthermore, sediment dynamic behaviour and ^137^Cs concentrations were expressed sufficiently well for each change in conditions (i.e., between dry and wet). The long-term estimations of the time-dependent behaviour of interspersed ^137^Cs indicated that the estimated ratio of decline in concentrations was slightly higher than could be explained by the redistribution processes in the Chernobyl region, indicating that the effects of the urban land cover were significant. The model also accurately reproduced the limited motion of fine particles in sediments of mixed grain size in medium- to small-scale floods, which was attributable to the shielding effect. Stream bed material did not move when the tractive force was less than a critical tractive value, which explains the near absence of the river flow effects of stream bed sediment and adsorbed ^137^Cs behaviour of the selected river channel. The context of sedimentation towards a downstream river construction structure (i.e., discharge) was also found to be a flushing effect (Phillips *et al*., (2000)^67^; Mouri *et al*., (2013a)^74^). Assessed values were generally in agreement with on-site observations of sediment and ^137^Cs concentrations. The vertical sections indicated not only the stream bed sedimentation form, but also the sediment ^137^Cs concentrations. A comparison of stream bed variation also confirmed the validity of the assessed results using the MLIT dataset. These differences were identified in the calculations performed for the selected stream bed near the estuary barrage and were discussed here by evaluating the validity of the model and its assessment. Observed vertical changes in the individual particle size distributions identified the validity of the model’s classification of the effects of the geomorphological condition of terraced land covered with volcanic ash soil on sediment and ^137^Cs dynamic behaviour (Erlandsson and Isaksson (2006)^28^; Mouri, (2014a)^1^). The results of these assessment improvements indicated that the grain sizes were reduced by the effects of low flow velocity, because of the role of the estuary barrage in inhibiting riverbed vertical variation. The agreement between calculated trends and observed phenomena further confirmed that the model correctly characterised the delivery of water, sediment and ^137^Cs into the selected typical urban rivers. The agreement of sedimentation and ^137^Cs concentrations, after incorporating the effects of mixed particle distributions and the assessed incoming and outgoing fluxes, with observational results confirmed the validity of the model. Furthermore, reductions in energy flow, river slope relief and sediment and ^137^Cs discharge in the low-gradient section at the time of a large flood event, as well as the ^137^Cs and sediment fluxes following fixation of unstable sediments due to estuary barrage effects, were demonstrated. Specifically, the assessment showed the amount of sediment and ^137^Cs passing through the estuary barrage structure, and accounted for water-vein factors, such as tractive force. The assessment method used in this study was limited in its ability to reproduce the complex phenomena occurring near the ^137^Cs control estuary barrage, incorporating the effects of radioactive decay, individual particle adsorption of ^137^Cs and its time-dependent behaviour, because stream bed changes were evaluated using only a one-dimensional model. The inventory of ^137^Cs and sediment concentrations indicated that deposition and discharge will decrease by 91–94% 50 years after the initial Fukushima accident. The model predicted that the regional riverbed variation, incorporating the effects of fine sediment particles as well as the effects of shell breaking during flood periods, will significantly affect the long-term radiological impact of ^137^Cs, compared to what would be predicted assuming circulation. The analyses indicated a large impact appearing during extreme flash flood events as ^137^Cs concentrations with incorporation of the amphibious effects of sediment particle adsorption in Tokyo as an urban region in Japan will likely decline faster than those observed in Chernobyl as a rural region.

This study provided more insight regarding this important topic than before the accident. The assessment identified the flux of interspersed sediment containing high concentrations of ^137^Cs from the estuary barrage incorporating the effects of extreme flash flood events. While this approach provided detailed geomorphological and hydraulic information, we also acquired coarse, multi-timescale information about the river channel by incorporating important landscape features into the assessment. These features were used to represent the ambient effects due to both soil and geomorphological characteristics in terraced land covered with volcanic ash soil incorporating the effects of ^137^Cs activity with sediment particle adsorption; therefore, it was possible to cover all of the important aspects of the cardinal river channel in this typical contaminated territory of the Tokyo region by selecting the appropriate assessment elements of ^137^Cs with incorporation of sediment particle adsorption to understand the environmental context and by tuning the related parameters to reflect their physical properties. The results of the sediment and ^137^Cs behaviours in terraced land covered with volcanic ash soil area were also assessed to understand the time-dependent behaviour in the selected catchment close to Mt. Fuji. The assessments showed that sediment and ^137^Cs concentrations declined faster than those observed in Chernobyl, with high concentrations of ^137^Cs in runoff during not only extreme rain events in the short-term, but also in the urban catchment over longer timescales (Mouri *et al*., (2014a)^1^, Mouri *et al*., (2014b)^75^; Erlandsson and Isaksson, (2006)^28^). Uncertainty, particularly regarding environmental and climate change, is represented by probabilistic linkages. The results of this study will be important to assess the dynamic behaviour of ^137^Cs with incorporation of the amphibious effects of sediment particle adsorption and the impact of time-dependent behaviour associated with ^137^Cs as one of the most important elements for both environmental and social contexts in terraced land covered with volcanic ash soil area close to volcanoes in different parts of the world.
